# DNA Damage Response and Oxidative Stress in Systemic Autoimmunity

**DOI:** 10.3390/ijms21010055

**Published:** 2019-12-20

**Authors:** Vassilis L. Souliotis, Nikolaos I. Vlachogiannis, Maria Pappa, Alexandra Argyriou, Panagiotis A. Ntouros, Petros P. Sfikakis

**Affiliations:** 1 First Department of Propaedeutic Internal Medicine and Joint Rheumatology Program, National and Kapodistrian University of Athens Medical School, 115 27 Athens, Greece; nivlachogiannis@gmail.com (N.I.V.); mariak.pappa@yahoo.com (M.P.); argyrioualex@gmail.com (A.A.); panagiotisd94@gmail.com (P.A.N.); psfikakis@med.uoa.gr (P.P.S.); 2 Institute of Chemical Biology, National Hellenic Research Foundation, 116 35 Athens, Greece

**Keywords:** DNA damage response and repair network, immune response, autoimmunity, systemic lupus erythematosus, systemic sclerosis, rheumatoid arthritis, oxidative stress, abasic sites, chromatin organization, apoptosis

## Abstract

The DNA damage response and repair (DDR/R) network, a sum of hierarchically structured signaling pathways that recognize and repair DNA damage, and the immune response to endogenous and/or exogenous threats, act synergistically to enhance cellular defense. On the other hand, a deregulated interplay between these systems underlines inflammatory diseases including malignancies and chronic systemic autoimmune diseases, such as systemic lupus erythematosus, systemic sclerosis, and rheumatoid arthritis. Patients with these diseases are characterized by aberrant immune response to self-antigens with widespread production of autoantibodies and multiple-tissue injury, as well as by the presence of increased oxidative stress. Recent data demonstrate accumulation of endogenous DNA damage in peripheral blood mononuclear cells from these patients, which is related to (a) augmented DNA damage formation, at least partly due to the induction of oxidative stress, and (b) epigenetically regulated functional abnormalities of fundamental DNA repair mechanisms. Because endogenous DNA damage accumulation has serious consequences for cellular health, including genomic instability and enhancement of an aberrant immune response, these results can be exploited for understanding pathogenesis and progression of systemic autoimmune diseases, as well as for the development of new treatments.

## 1. Introduction

The human genome confronts thousands of DNA lesions every day due to normal “mistakes” during DNA replication, or exposure to exogenous or endogenous “toxic” factors, which can block the replication process, lead to genomic instability, and threaten cell function and homeostasis [1]. To ensure proper cell function and viability, a well-organized mechanism, namely, DNA damage response and repair (DDR/R) network, has been evolved over the years. DDR/R is a hierarchically structured mechanism, the main aspects of which are conserved from prokaryotes and phages to humans [2], consisting of sensors, mediators, transducers, and effectors, which recognize any defects during the cell cycle and assign the proper repair process [1]. In case of unrepaired lesions and depending on the extent and type of damage, the cell either passes the mutated genome to its offspring or is neutralized by programmed cell death (apoptosis) or senescence [2].

The interplay between DDR/R and innate immune response has been increasingly recognized in the past years. Several studies have demonstrated that a shift in the balance of DDR/R network driven by either exposure to DNA-damaging agents or deregulation of DNA repair mechanisms results in the accumulation of cytosolic single-stranded DNAs (ssDNAs) and double-stranded DNAs (dsDNAs) that can act as potent immunostimulators through the induction of the cGAS-STING (stimulator of interferon genes)-IRF3 pathway and the production of type I interferon (IFN) [3,4,5,6,7,8,9,10,11]. Moreover, recent studies have shown that cell cycle progression through mitosis following DNA double-strand breaks (DSBs) formation and DDR/R induction leads to the generation of micronuclei, which precede activation of the immune system [12,13,14]. On the other hand, loss of immune homeostasis and prolonged inflammatory response generated by different sources (infection, radiation, toxins, autoimmunity, ageing, etc.) can lead to DNA damage and activate the DDR/R network [15,16,17,18,19,20,21], proposing a bi-directional relationship between DDR/R and immune response (ImmR) [2].

Systemic autoimmune disorders comprise a heterogeneous group of diseases characterized by aberrant immune response to self-antigens with widespread production of autoantibodies and multiple tissue injury, as well as by oxidative stress along with the excess production of reactive oxygen species (ROS) and reactive nitrogen species (RNS). Inappropriate activation of adaptive immunity and production of autoantibodies has been classically linked to autoimmunity, whereas innate immune activation and, specifically, the recognition of nucleic acids by Toll-like receptors and other cytoplasmic innate immune receptors, are considered as part of the pathophysiology of autoimmune diseases [22].

Aberrant DDR/R has been reported in patients with systemic autoimmune diseases, such as systemic lupus erythematosus (SLE) [3,4], systemic sclerosis (SSc) [23], and rheumatoid arthritis (RA) [24,25]. Polymorphisms of nucleases or molecules central in the DNA repair process have been detected with increased frequency among patients with autoimmune diseases, whereas gene/protein expression assays have shown downregulation of molecular components that are implicated in the DNA repair machinery and upregulation of apoptosis genes among patients with autoimmune disease [4]. However, only a few studies to date have examined the burden of DNA damage in patients with systemic autoimmune diseases and mechanistic aspects underlying this phenomenon. Whether aberrant DDR/R response precedes immune activation in autoimmune diseases or the chronic immune activation/inflammation leads to increased DNA damage formation and deregulation of DNA repair mechanisms remains largely unknown. Recently, our group suggested a role of deficient DNA repair and increased formation of endogenous DNA damage, at least partly due to the induction of oxidative stress, that may lead both to augmented apoptosis rates and subsequent autoantibody production in patients with SLE [3,4].

In the current review, we first briefly overview the normal DDR/R pathways and highlight DDR/R aberrations and critical endogenous factors/processes that lead to the intracellular formation of DNA damage, which are observed in systemic autoimmune diseases. The main goal of this review is to serve as a “tool” for the comprehensive presentation of up-to-date literature on the subject and thus help in the design of new mechanistic studies to better understand the involvement of the DDR/R network in the pathogenesis of systemic autoimmunity, as well as to suggest new therapeutic perspectives and potential targets.

## 2. Normal DNA Repair Pathways

To compensate for the many types of DNA damage that occur, cells have developed multiple repair mechanisms wherein each corrects a different subset of lesions. In general, there are six major DNA repair pathways, which will be presented below.

### 2.1. Nucleotide Excision Repair (NER)

NER is a fundamental DNA repair mechanism involved in the removal of bulky, helix-distorting lesions from DNA [26]. DNA adducts that are repaired by NER include cyclobutane pyrimidine dimers (CPDs) and 6-4 photoproducts (6-4 PPs) produced by UV radiation, DNA lesions generated by ROS or endogenous lipid peroxidation products, intrastrand cross-links and adducts produced by genotoxic drugs (melphalan, cisplatin), or environmental carcinogens (benzo[a]pyrene) [27,28]. There are two subpathways of NER, termed GGR (global genome repair) and TCR (transcription-coupled repair), where approximately 30 proteins are involved in both subpathways. The first step, the recognition of DNA damage, differs between the two subpathways. In GGR, the formation of a bulky DNA adduct induces an increase in helix distortion, which facilitates the recruitment of the damage recognition factor XPC/RAD23/CEΤN2 and UV-DDB. On the other hand, damage recognition in TCR is initiated when an elongating RNA polymerase II (RNAPII) is arrested upon encountering a site of DNA damage. Subsequently, two TCR-specific proteins, Cockayne syndrome A (CSA) and B (CSB), are thought to displace the stalled RNAPII to allow the access of the NER proteins to the lesion. Following damage recognition, both GGR and TCR proceed through common NER reactions. The biological importance of NER for human health is obvious by the fact that defects in this repair pathway cause several human genetic disorders, including Cockayne syndrome (CS), xeroderma pigmentosum (XP), and trichothiodystrophy (TTD), which are all associated with photosensitivity [29].

### 2.2. Base Excision Repair (BER)

BER is a conserved and ubiquitous DNA repair pathway, which recognizes and removes damaged DNA bases that do not significantly distort the structure of the DNA helix [30]. BER is used by the cell to correct DNA lesions that occur through the spontaneous deamination or hydroxylation of bases and by oxidation of nucleotides by ROS produced either by normal metabolism or environmental stresses such as smoking, oxidizing chemicals, or ionizing radiation [31]. In addition, BER is implicated in the repair of alkylated DNA bases generated by endogenous or exogenous factors (carcinogens, antineoplastic drugs, etc.), which if left unrepaired produce mutations in the cells [32]. BER consists of two subpathways, known as single-nucleotide or short-patch and long-patch; the activation of one or the other is predicated by the cause and type of damage, the type of abasic (AP; apurinic/apyrimidinic) site generated in the first repair step and the cell cycle phase in progress when the damage occurs. The short-patch pathway quickly repairs single-base damage during the G1 phase; the long-patch pathway handles lengthier repair during S or G2, when resynthesis of two to eight nucleotides surrounding the AP-site is required. Among the enzymes that take part in BER, DNA glycosylases, mono- or bi-functional, are the most important. They recognize and hydrolyze the *N*-glycosylic bond between the damaged base and the sugar phosphate backbone, creating an AP intermediate site.

### 2.3. Mismatch Repair (MMR)

MMR mechanism is a major contributor to replication fidelity, which removes base substitution and insertion/deletion mismatches that arise as a result of replication errors escaping the proofreading function of DNA polymerases [33]. The recognition of DNA lesions is accomplished by the complex Mutator Sα (MUTSα), a heterodimer of the DNA mismatch repair proteins Mutator S homolog 2 (MSH2) and Mutator S homolog 6 (MSH6). Another heterodimer complex, called MUTSβ, which consists of MSH2 and MSH3, is able to bind only to insertion/deletion mismatches. Lesion recognition is followed by the recruitment of Mutator Lα (MutLα) [MLH1/postmeiotic segregation increased 2 (PMS2)] or MutLβ (MLH1/MLH3), which have endonuclease activity that can incise DNA near the mismatch. The nick is used by the 5′ exonuclease 1 (Exo1) as an entry point to degrade DNA past the mismatch, and the resulting single-stranded DNA gap is filled in by polymerase δ and sealed with DNA ligase I [34,35]. Deficiencies in MMR lead to microsatellite instability (MSI), which is a pattern of hypermutation that occurs at genomic microsatellites, and is associated with unique clinical features, prognosis and response to therapy, and immune checkpoint blockade [36,37].

### 2.4. Double-Strand Breaks (DSBs) Repair

DSBs may occur as a result of exposure to both exogenous factors, including ionizing radiation, UV light and genotoxic drugs [38], and endogenous events, including oxidative stress, replication fork collapse, and telomere erosion [39]. Of interest, these lesions also occur as programmed events during meiosis, as well as during V(D)J recombination [the process by which T cells and B cells randomly assemble different gene segments—known as variable (V), diversity (D) and joining (J) genes—in order to generate unique receptors (known as antigen receptors) that can collectively recognize many different types of molecule] and class-switch recombination (CSR) required for immunoglobulin diversity and function [40]. DSBs, if left unrepaired, have severe adverse consequences for the cell including the generation of mutations, chromosomal aberrations, and cell death [41]. To maintain genomic integrity, cells have evolved several pathways to remove DSBs.

#### 2.4.1. Homologous Recombination Repair (HRR)

HRR is an error-free DNA repair mechanism, which operates during the S and G2 phases of the cell cycle so that it can find a large area of homology on a sister chromatid to use as a template for resynthesizing damaged or lost bases [42]. HRR can be divided into several steps. During initiation, both the 5′-ends of the DSB are resected by the action of a specific nuclease to yield 3′-single-strand DNA (3′-ssDNA) tails. Then, one of these tails invades an intact homologous duplex and generates a D-loop structure, while the other could simply anneal with the displaced strand at the joint. Both 3′-ends then prime new DNA synthesis using the intact duplex as a template. This process, followed by ligation, leads to the formation of two Holliday junctions (four-stranded branched structures), which are finally cleaved by the action of a resolvase [43].

#### 2.4.2. Canonical Non-Homologous End Joining (c-NHEJ)

c-NHEJ is an error-prone process, which is active throughout the entire cell cycle. C-NHEJ is initiated by the binding of the heterodimeric protein complex X-ray repair cross complementing 5/6 to both DNA ends. Then, DNA-dependent protein kinase (DNA-PK) is recruited, a DNA dependant protein kinase, which activates X-ray repair cross-complementing protein 4 (XRCC4)-ligase IV complex to link the broken DNA ends together. However, before re-ligation, MRN complex (MRE11-Rad50-NBS1), together with the Flap Endonuclease 1 (FEN1) and Artemis, are involved in processing DNA ends [44,45]. Aberrant c-NHEJ is a major source of genomic rearrangements and chromosomal translocations, leading to genomic instability [46]. Interestingly, deficient c-NHEJ is associated with defective V(D)J recombination and immune defects [47]. The choice between c-NHEJ and HRR pathways is regulated by complex regulatory mechanisms and involves competition between the p53-binding protein 1 (53BP1), which favors c-NHEJ, and BReast CAncer gene 1 (BRCA1), which promotes HRR. Methylation of histone H4 by Multiple Myeloma SET (MMSET) results in 53BP1 recruitment at the DSB site, which blocks DNA end resection by the MRN complex, C-terminal binding protein 1 interacting protein (CtIP), and BRCA1. On the other hand, histone H4 acetylation by the Tat-interactive protein (Tip60) blocks 53BP1 recruitment and promotes BRCA1 occupancy and HRR. Cell cycle-regulated proteins such as cyclin-dependent kinases also play a key role in the choice of pathway to resolve DSBs [38].

#### 2.4.3. Alternative Non-Homologous End Joining (alt-NHEJ)

alt-NHEJ is a mechanistically distinct pathway of DSB repair that is frequently termed microhomology-mediated end-joining [48]. Indeed, the foremost distinguishing property of alt-NHEJ is the use of 5–25 base pair microhomologous sequences during the alignment of broken ends before joining, thereby resulting in deletions flanking the original break [49]. Thus, alt-NHEJ is frequently associated with chromosome abnormalities including translocations, deletions, and inversions [50]. The viewpoint that alt-NHEJ is the major DNA repair pathway to pathogenic chromosomal errors is further strengthened by the finding that c-NHEJ-deficient mice develop tumors with chromosomal translocations generated by alt-NHEJ [51].

#### 2.4.4. Single-Strand Annealing (SSA)

SSA is a highly mutagenic but very efficient DSB repair mechanism [38,52]. This process involves a DSB between homologous repeats, followed by DSB end resection that generates 3′-ssDNA, which reveals flanking homologous sequences that are annealed together to form a synapsed intermediate. This intermediate is then processed for ligation, which requires endonucleolytic cleavage of nonhomologous 3′-ssDNA tails, and polymerase filling of the gaps. Genetically, SSA is distinct from other homologous recombination pathways, as it occurs independently of Rad51 recombinase. Instead, it depends on Rad59, the Rad52 paralog that is structurally homologous to the N-terminus of Rad52. Biochemically, both Rad52 and Rad59 can anneal ssDNA, but only Rad52 can anneal ssDNA coated with RPA proteins. Although relatively mutagenic in terms of causing a rearrangement between repeat elements, SSA is critical to restore a broken chromosome with DSB ends that have undergone extensive end resection, but are unable to be resolved by HRR or alt-NHEJ [53]. The importance of SSA in DNA repair depends on a number of factors, including the state of the cell cycle, the presence or absence of the sister chromatid, and the length of uninterrupted homology.

### 2.5. Interstrand Cross-Link (ICL) Repair

The formation of cross-links between the two strands of DNA is considered a critical event, causing cell cycle and replication arrest and eventually cell death if not repaired [54]. Cross-linking agents are exogenous chemicals, including the drugs cyclophosphamide, melphalan, cisplatin, mitomycin C, and psoralen [55], as well as endogenously formed aldehydes [56]. There are three routes for cross-link detection in mammalian cells. Adducts can be recognized in otherwise unperturbed duplex DNA by factors that recognize DNA damage. Cross-link detection might also occur via encounter with the transcription machinery. Finally, ICLs could block a replication fork, triggering a repair response that would remove the cross-link and restore replication. Interestingly, in non-replicating cells, the repair of ICL is mediated by the NER mechanism and by the DNA translocase FANCM, which facilitates the access of nucleases to the lesion. In S-phase cells, cross-link repair is coupled to DNA replication, features DSBs as repair intermediates, and depends on the homologous recombination machinery [57]. ICL repair in human cells is accomplished in four distinct steps: (a) unhooking of the ICL on one strand and induction of a DNA replication-dependent DSB, (b) translesion DNA synthesis using the DNA strand with the unhooked ICL as a template, (c) processing of the DSB and restoration of the stalled DNA replication fork, and (d) removal of the residual unhooked ICL [58]. Proteins implicated in the repair of ICLs have a critical role in the pathophysiology of several hereditary disorders, such as Fanconi anemia, xeroderma pigmentosum, Cockayne syndrome, cerebro-oculo-facio-skeletal syndrome, and trichothyodistrophy.

### 2.6. Direct Repair Pathway

The direct repair mechanism is a single step pathway, which is unique in that only one protein is implicated in the repair process [59]. Indeed, the sole protein involved, O6-methylguanine-DNA methyltransferase (MGMT), removes alkyl groups from the O6 position of guanine or to a lesser extent from the O4 position of thymine, such as those generated by treatment with alkylating drugs (procarbazine, dacarbazine, temozolomide), and transfers it to an internal cysteine residue of MGMT [60]. Because the alkyl group is covalently bound to the MGMT protein, MGMT is functionally inactivated after each reaction, and degraded through the ubiquitin proteolytic pathway. Without MGMT repair, alkyl adducts would cause thymine mispairing during replication, leading to G:C to A:T transitions or strand breaks [61]. Overactivity of MGMT is also considered responsible for chemoresistance; for example, >90% of recurrent gliomas show no response to a second cycle of chemotherapy. Conversely, inhibition of MGMT renders cancer cells sensitive to temozolomide, whereas MGMT promoter alkylation is a significant determinant in the sensitivity of drugs such as temozolomide. There is abundant evidence linking methylation of the MGMT promoter to loss of protein expression, resulting in increased sensitivity to chemotherapeutic agents and to the prognostic outcome of patients treated. Similarly, low MGMT expression appears to be a biomarker for slower tumor progression [62].

## 3. The Interplay between the DDR/R Network and the Immune Response: The Role of Oxidative Stress

Although not completely delineated to date, interplay between the DDR/R network and the ImmR has been suggested by a series of studies, nicely reviewed in [2]. A first hint that defective nucleic acid metabolism may trigger aberrant innate immune activation with serious consequences for life is derived from Aicardi-Goutières (AGS) syndrome. AGS is a childhood-onset, possibly fatal encephalopathy characterized by mutations of central molecules implicated in DNA and RNA metabolism, such as (a) RNaseH2, which is involved in excision of a single ribonucleotide embedded in genomic DNA and removal of an R-loop formed in cells [63,64,65], or (b) the 3′–5′ exonuclease TREX1 [66,67,68]. On the other hand, increasing data suggest that aberrant, chronic (auto)immune activation and chronic inflammation may cause DNA damage and trigger the DDR/R network [4]. Indeed, under inflammatory conditions, ROS and RNS are generated from inflammatory and epithelial cells and result in oxidative and nitrative DNA damage, such as 8-oxo-dG and 8-nitro-dG, as well as in the inhibition of key proteins of the DNA repair machinery, indicating the bi-directional interplay between DDR/R and ImmR via oxidative stress [2].

The central role of type I IFN pathway activation in the pathophysiology of systemic autoimmune diseases has been extensively studied since its first description almost 40 years ago [69,70]. The recognition of nucleic acids by innate immune receptors (Toll-like receptors (TLR) and non-TLRs) has a central role in autoimmunity, suggesting that abnormal DNA or RNA metabolism may initiate and/or perpetuate innate immune activation [71]. Innate immune receptors can either recognize pathogen-derived “non-self” DNA (pathogen-associated molecular patterns, PAMPs), for example, those derived from a DNA virus, but also damaged “self” DNA (damage-associated molecular patterns, DAMPs) at sites of inflammation, and can initiate an immune response [2]. In physiological conditions, DAMPs are found intracellularly; are invisible to the immune system; and serve metabolic, structural, or enzymatic functions [72]. On the other hand, DAMPS are exposed or released upon stress, injury, and cell death, thereby becoming able to bind appropriate receptors on immune cells. Of note, following treatment with some anticancer drugs, such as anthracyclines (doxorubicin, epirubicin, idarubicin), mitoxantrone, oxaliplatin, cyclophosphamide, and bortezomib, cancer cells undergo a form of cell death named immunogenic cell death, which is characterized by an increased immunogenic potential, owing to the emission of the DAMPs, which act as danger signals to produce immunostimulatory effects, such as the recruitment and activation of neutrophils, macrophages, and other immune cells [73]. DAMPs released during immunogenic cell death include plasma membrane exposure of endoplasmic reticulum chaperones such as calreticulin (CALR), secretion of ATP, release of double-stranded DNA resulting in activation of STING and release of type I IFN and proinflammatory cytokines, secretion CXCL10, as well as the release of high-mobility group box 1 (HMGB1) and annexin A1 (ANXA1) [74].

### 3.1. DNA Double-Strand Breaks Per Se Induce Innate Immune Activation

Apart from oxidative DNA damage, the presence of DSBs *per se* has been shown to induce type I IFN production [5]. Indeed, treatment of healthy donor-derived primary monocytes with etoposide, a chemotherapeutic agent that blocks topoisomerase II activity and leads to the accumulation of DSBs, up-regulated type I IFN-induced gene expression and type III IFNs (IFN-λ). Similarly, other DSB-inducing drugs (mitomycin C, adriamycin, etc.) were also able to induce type I and III IFNs in primary monocytes and various cell lines, suggesting that DDR-induced IFN expression is a universal mechanism that may underline different pathological processes [5]. In line with these results, treatment of breast cancer cell lines with DSB-inducing drugs, including ionizing radiation or therapeutic drugs (mitomycin C, cisplatin), led to the accumulation of cytoplasmic ssDNA and finally the activation of the STING-IRF3 pathway [10]. Furthermore, basic components of DSB repair were shown to be responsible for the production of cytoplasmic ssDNA, which seems to be the main immunostimulant [10]. Interestingly, TREX1 was found to be the main responsible nuclease for the restriction of this cytoplasmic ssDNA and prevention of aberrant innate immune activation [6,10]. An association between immune activation and TREX1 was also observed when TREX1 null mice developed inflammatory myocarditis due to an interferon-dependent autoimmune response leading to dilated cardiomyopathy and a significantly reduced survival [75]. Of note, type I IFN is indeed implicated in the inflammatory myocarditis and early mortality observed in this mouse model, as disease manifestations were strikingly attenuated in Trex1-deficient mice also lacking the type I IFN receptor (IFNαR1) [76], and were also improved in mice treated with an inhibitor of the downstream kinase TANK-binding kinase 1 [77].

### 3.2. Defective DNA Repair and Chronic Low-Level DNA Damage “Prime” the Innate Immune Response

**Figure 1:**
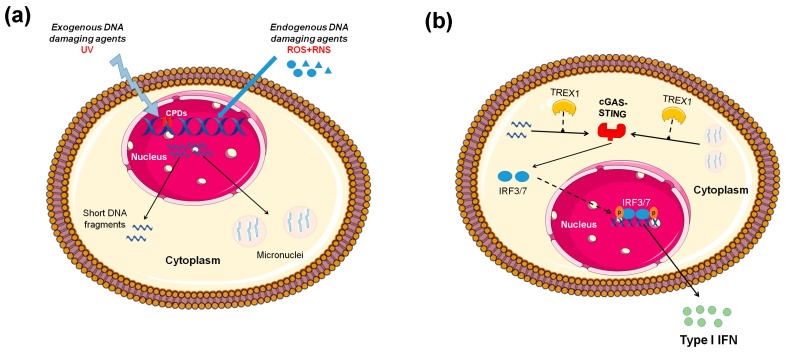
Induction of type I interferon (IFN) expression by endogenous DNA damage. (**A**) Exogenous and/or endogenous genotoxic agents may lead to the accumulation of DNA damage in the nucleus, followed by exportation of damaged DNA into the cytoplasm and the induction of micronuclei. (**B**) Damaged cytoplasmic DNA, if it is not cleared by the exonuclease Trex1, activates the cGAS-STING (stimulator of interferon genes)-IRF3 pathway and the production of type I IFN. ROS: reactive oxygen species, RNS: reactive nitrogen species, CPDs: cyclobutane pyrimidine dimers.

Another clue that defective DNA repair primes innate immune response comes from ataxia–telengiectasia (AT), a neurodegenerative disorder associated with mutations of the central DNA repair kinase ATM [6]. In this study, Härtlova and colleagues showed that AT-derived fibroblasts had higher constitutive expression of type I and III IFNs and mounted a profoundly high response upon transfection with DNA virus or the intracellular microbe *Listeria* monocytogenes. These results suggested that loss of ATM, potentially leading to chronic accumulation of low-grade DNA damage, may prime the innate immune response [6]. Similar results were obtained from ATM-deficient mice and cell lines where ATM was silenced. Of interest, γ-irradiation or etoposide treatment of normal bone marrow-derived myeloid cells mimicked the elevated basal expression of IFN and hyper-sensitivity to PRR (TLR and non-TLR)-induced response observed in ATM-deficient cells, suggesting that the accumulation of damaged DNA underlined this phenomenon. A series of mechanistic studies with knock-out of various innate immune adaptors revealed that STING was mainly responsible for the observed phenotype [6]. In summary, ATM deficiency led to the accumulation of DNA damage, exportation of damaged ssDNA and dsDNA into the cytoplasm, activation of the cGAS-STING pathway, and finally type I IFN production that primed cells for response to exogenous or endogenous stimuli such as viral or bacterial infections (Figure 1)**.**

Moreover, Günther and colleagues suggested that defective ribonucleotide removal and accumulation of base lesions and low-grade DNA damage “primed” ImmR [69]. Indeed, they showed that fibroblasts from AGS and SLE patients with mutations in the DNA repair enzyme RNaseH2 produced increased levels of IFNβ upon stimulation with poly(I:C), a phenomenon that was enhanced when poly(I:C) treatment was combined with UVC irradiation. In the same study, patients’ fibroblasts showed a decreased proliferation rate in vitro, increased p53 phosphorylation at Ser15, and senescence. Of interest, RNaseH2 deficiency in heterozygous carriers (parents of AGS patients) significantly increased the prevalence of antinuclear antibodies (ANAs), suggesting that defective ribonucleotide removal may promote formation of autoantibodies [69]. Type I IFN activation in RNaseH2-null cells was also shown to be mediated by STING [11]. That is, dermal fibroblasts isolated from AGS/SLE patients with RNaseH2 mutations and mouse embryonic fibroblasts (MEFs) isolated from RNaseH2-null mice showed significantly increased single-strand breaks (SSBs) and DSBs and were also more sensitive to UV-irradiation, as shown by increased CPD formation.

### 3.3. Micronuclei: Connecting Nuclear DNA Damage and Cytosolic Innate Immune Receptors

The strict compartmentalization of DNA in the cell’s nucleus and mitochondria raises the question as to how damaged self DNA becomes accessible to STING, which resides in the cytoplasm. Recent sophisticated studies connected the dots featuring a new role for micronuclei [70]. Micronuclei are components of the nuclear membrane encompassing DNA, which are released in the cytoplasm during mitotic cell division. Two independent studies showed that RNaseH2-null cells, which have been previously shown to express higher levels of IFN-induced genes, probably through a STING-mediated pathway [11,69], have increased numbers of micronuclei in their cytoplasm [12,14]. Of note, the majority of micronuclei were enriched for cGAS, which is essential for the production of cGAMP, the activator of STING [71].

### 3.4. Oxidative Stress Causes DNA Damage That Activates the Immune System

Cellular oxidative damage is a general mechanism of cell and tissue injury, which is primarily caused by free radicals and ROS. ROS are chemically reactive molecules containing an oxygen atom. Although normally ROS are essential elements of the ImmR involved in cytokine production, microbial clearance, cell proliferation, and cell death, overproduction and/or inadequate removal of these species results in oxidative stress [78].

ROS are produced by both endogenous and exogenous sources. Endogenous sources include the generation of ROS from mitochondria; peroxisomes (intracellular organelles that are also called microbodies); activated inflammatory cells, such as macrophages, neutrophils, and eosinophils; as well as during the metabolism of xenobiotics mediated by cytochromes P450 oxidoreductases [79]. These endogenously induced DNA lesions can often reach a level much higher than the ones induced by environmental factors. ROS are constantly generated in mitochondria as respiration byproducts (1–5% of consumed oxygen), and in general are accepted as the major source of oxidative injury in aerobic organisms. Another source of constant generation of free radicals is the chronic exposure to viral infections. The high intracellular oxidation status in viral infections consists of decreased antioxidant enzymes such as catalase, glutathione peroxidase, glutathione reductase, as well as high levels of hydroxyl radicals. Of note, tumor growth and development is always accompanied by oxidative stress, which develops due to various inflammatory and immune reactions [80].

As for the extracellular sources of ROS, these include ionizing radiations such as X-, γ-, or cosmic rays and α-particles from radon decay, oxidizing chemicals, ultraviolet A (UVA) light, chemotherapeutics, environmental toxins, and other pollutants [78]. Exposure to extracellular sources of ROS is especially prevalent in skin cells, as they are constantly exposed to the environment. Radiation can react with oxygen and form superoxide anion radical, hydroxide anion, and hydroxyl radical that are able to destroy the structural integrity of DNA. Moreover, chronic exposure to cigarette smoke promotes lipid peroxidation and has detrimental effects for the cardiac and respiratory systems. Some xenobiotics appear to interfere with mitochondrial bioenergetics and promote superoxide production.

DNA lesions associated with ROS are oxidized purines and pyrimidines, SSBs, DSBs, and abasic sites. Two of the most common endogenous DNA base modifications are 8-oxo-7,8-dihydroguanine (8-oxoGua) and 2,6-diamino-4-hydroxy-5-formamido-pyrimidine. These lesions can be originated from the addition of the hydroxyl radical to the C8 position of the guanine ring producing a 8-hydroxy-7,8-dihydroguanyl radical, which can be either oxidized to 8-oxoGua or reduced to give the ring-opened 2,6-diamino-4-hydroxy-5-formamidopyrimidine (FapyGua) [81]. Moreover, interaction of hydroxyl radical with pyrimidines (thymine and cytosine) at positions 5 or 6 of the ring can produce several base lesions, such as 5,6-dihydroxy-5,6-dihydrothymine and 5,6-dihydroxy-5,6-dihydrocytosine. Two other pyrimidine lesions are the 5-(hydroxymethyl) uracil and the 5-formyluracil, which are often detected in humans as the result of the interaction of the hydroxyl radical with the methyl group of thymine. With the interaction of the hydroxyl radicals with DNA, SSBs may also occur, which in turn trigger the induction of DSBs. The mechanism consists of hydrogen abstraction from the 2-deoxyribose, leading to the formation of carbon-based radicals, which under the presence of oxygen can be converted to peroxyl radicals. The peroxyl radicals, through different reactions, can also abstract hydrogen atoms from sugar moieties, thus leading to DNA strand breaks. The most prevalent and characteristic abasic sites formed under oxidative stress are 2-deoxyribonolactone and the C4′ oxidized abasic site that arise from hydroxyl radical-mediated hydrogen abstraction at C1 and C4 of the 2-deoxyribose moiety of DNA, respectively [82]. Interestingly, peroxyl radical-mediated DNA adducts are potential precursors of apurinic sites, as the opening of the imidazole ring of the purine bases may lead to increased hydrolytic lability of their *N*-glycosidic bonds. This occurrence is very common and can occur spontaneously or enzymatically as “repair intermediates” of the BER pathway.

On exposure to oxidative stress, cells initiate a variety of defense mechanisms, including both enzymatic and non-enzymatic antioxidants. In mammalian cells, enzymatic antioxidants include superoxide dismutase, glutathione peroxidase, glutathione reductase, glutathione-S-transferase, and catalase, whereas non-enzymatic antioxidants contain ascorbic acid (vitamin C), α-tocopherol (vitamin E), total thiol, glutathione, carotenoids, and flavonoids [83]. Oxidative stress causes damage on the primary cellular components, including DNA, proteins, and lipids. In particular, ROS-induced DNA lesions include oxidized bases, abasic sites, single-strand breaks (SSBs), and DSBs, which during the replication process can lead to replication fork stalling, thus giving rise to mutations and genetic instability [84].

Accumulating evidence suggests that oxidative stress can participate in the pathogenesis, progression, and complications of many diseases, including cancer and autoimmunity [78]. Especially with regard to systemic autoimmune diseases, several studies have shown that SLE patients are characterized by increased oxidative stress, resulting in immune system dysregulation, abnormal activation and processing of cell-death signals, and autoantibody production [85]. Indeed, previous studies have shown that oxidative stress causes a significant delay in the apoptotic clearance, resulting in a prolonged interaction between ROS and nuclear residues, which in turn triggers neo-epitope production and autoantibody formation [86]. In addition, oxidative stress is involved in the pathogenesis of SSc [87]. That is, SSc patients are characterized by increased production of ROS in the skin, visceral fibroblasts, and endothelial cells, as well as by reduced concentrations of various antioxidants, including antioxidant vitamins (ascorbic acid, α-tocopherol, β-carotene) and minerals (zinc, selenium) [88]. Also, oxidative stress has been observed in patients with RA. In fact, these patients show augmented intracellular ROS, lipid peroxidation, protein oxidation, DNA damage, and deregulated antioxidant defense system of the body. Moreover, deficient MMR system was observed in RA patients, resulting in increased formation of DNA adducts in the joints and acceleration of the disease progression [89]. In line with these data, low levels of non-enzymatic antioxidants [reduced glutathione (GSH) and vitamin C] were found in RA patients, as compared with healthy individuals.

In an attempt to explain the immunogenicity of “self” DNA under inflammatory conditions, Gehrke and colleagues used a series of in vitro experiments and revealed a role for oxidized DNA as DAMP [90]. Indeed, they found that oxidized DNA, that is, from UV-induced damage or from ROS released during cell death, activated the innate immune receptor STING (stimulator of interferon genes), whereas “normal” DNA did not. Further exploring the mechanistic aspects of this “paradox”, oxidized DNA was shown to be resistant to TREX1 degradation, thus accumulating in the cytoplasm and activating the cGAS-STING and the type I IFN pathway. Of note, this axis, that being oxidized DNA-cGAS-STING-type I IFN, was verified in a SLE mouse model [Murphy Roths Large lymphoproliferation (MRL/lpr) mice], as well as in skin biopsies of patients with SLE, where oxidized DNA co-localized with the type I IFN-induced gene myxovirus (influenza) resistance 1 (MX1) [90].

The immunogenicity of UV radiation and subsequent oxidative DNA damage, as observed in SLE flares following sun exposure, has also been studied in preclinical models [91]. The researchers showed that UV radiation potentiates STING-dependent activation of IFN regulatory factor 3 (IRF3; immune signaling transcription factor) in response to cytosolic DNA and cyclic dinucleotides in keratinocytes and other human cells. Furthermore, they found that stimulation of STING-dependent IRF3 by UV is due to apoptotic signaling-dependent disruption of ULK1 (Unc51-like kinase 1), a pro-autophagic protein that negatively regulates STING.

### 3.5. Oxidative Stress and Immune Senescence

It is generally accepted that oxidative stress induces the senescent phenotype. Cellular senescence is a cell state implicated in various physiological processes and a wide spectrum of age-related diseases [92]. There are four different molecular mechanisms of oxidative stress-induced cell senescence: (a) the DDR/R mechanism, in which oxidative damage stimulates the DDR/R network through activating p53 and up-regulating p21 expression to cause senescence [93]; (b) the nuclear factor kappa B (NF-κB) mechanism, in which oxidative stress activates the inhibitor of kappa B (IκBs) kinase, which phosphorylates IκB to activate NF-κB and makes it transfer into the nucleus to stimulate IL-8 expression and increase p53 protein stability and then induce cellular senescence [94]; (c) the p38 mitogen-activated protein kinase (MAPK) mechanism, which is activated by ROS, up-regulates p19 protein expression, and limits self-renewal (the process by which stem cells divide to make more stem cells) to induce cellular senescence [95]; and (d) the microRNA mechanism, in which oxidative stress affects the amount of microRNA and promotes senescence [96].

Interestingly, the senescence-induced decline of the immune system is known as immunosenescence and is implicated in impaired autoantigen recognition and vaccination in the elderly. Indeed, several functions of the cells involved in the innate and adaptive immune responses are seriously compromised with age progression, including chronic inflammatory state, changes in lymphocyte subsets, and decreased proliferative responses, among others. Recent data have shown that during senescence, the LINE-1 retrotransposon is transcriptionally expressed and stimulates the IFN-I response, thus contributing to the maintenance of the senescence-associated secretory phenotype, which determines the ability of senescent cells to express and secrete cytokines, chemokines, proteases, growth factors, and bioactive lipids [97].

Moreover, age-related transformations redesign the immune architecture and the balance between pro-inflammatory and anti-inflammatory protective factors, as well as between pro-apoptotic and anti-apoptotic signals. In fact, elderly people experience increased reactivity to autoantigens, loss of tolerance, and systemic inflammation, while at the same time they suffer from degenerative diseases, which, in turn, increase the risk of developing an autoimmune disease [98]. Moreover, epigenetic changes and the increase in inflammatory cytokines and chemokines such as TNF-α, C-reactive protein, IL-8, MCP1, and RANTES (Regulated on Activation, Normal T Cell Expressed and Secreted) that occur in the elderly play a crucial role in the onset of autoimmune diseases [99].

These alterations make older persons more prone, not only to autoimmune disease, but also to cancer, as well as metabolic, neurodegenerative, and infectious diseases [100]. In fact, infectious diseases account for roughly 20% of hospitalizations in the elderly, whereas one-third of deaths in persons aged >65 years has been reported to be due to infectious diseases [101]. In addition, immunosenescence also results in reduced responses to vaccination, a common phenomenon in the elderly [102] Growing interest in therapeutically targeting senescence to improve healthy aging and age-related disease with compounds known as senolytic drugs has recently led to the first clinical trials [92].

### 3.6. Defects in Degradation of Endogenous DNA and Immune Activation

Apart from increased DNA damage formation and defective repair, a third mechanism may also be implicated in the accumulation of immunogenic damaged DNA—defective DNA degradation. Indeed, degradation of cytosolic DNA by TREX1 is integral for the prevention of aberrant innate immune responses [76]. Although previous mechanistic studies have revealed TREX1 as the main nuclease for removal of oxidized DNA, DNAse II may also have an important role in prevention of misplaced innate immune response, as DNAse II-deficient mice have been shown to spontaneously develop polyarthritis mimicking RA [103].

## 4. The DDR/R Network in Systemic Autoimmune Diseases

### 4.1. Systemic Lupus Erythematosus

SLE is a prototypic autoimmune disease characterized by abnormal T and B cell responses, in which excessive antibody production and immune complex formation are considered central pathogenetic mechanisms [104]. In the past years, innate immunity and specifically the recognition of nucleic acids by TLRs and cytoplasmic receptors have also been gaining attention as critical components in SLE pathogenesis [105]. The first hint that abnormalities in DDR/R pathway may be involved in SLE pathophysiology comes from the increased frequency of polymorphisms of central molecules involved in the DDR/R pathway such as TREX1 [106]. Moreover, autoantibodies against components of the DDR pathway have been detected in approximately 10%–20% of patients with SLE [107]. Deficiencies in DNA repair have been shown to induce lupus-like disease in animals. Mice carrying the Y265C hypomorphic allele of POLB (DNA polymerase, beta; a key enzyme in BER mechanism) demonstrated several pathologies resembling lupus, such as nephritis and skin manifestations, along with high titers of anti-nuclear antibodies in serum [108]. Moreover, mice with compound deficiency in Gadd45β (Growth arrest and DNA-damage-inducible, beta) and Gadd45γ proteins, which are involved in DDR/R as well as in initiation of the type 1 helper T cell (Th1) response, showed features resembling lupus, such as antibodies against dsDNA and histones in sera, and immune complex deposits in renal glomeruli [109].

#### 4.1.1. Gene Polymorphisms Associated with Impaired DNA Repair Machinery and SLE

Data from two cohort studies evaluated the association of the most common polymorphisms of XRCC1, a ligase protein involved in BER, with SLE susceptibility. The rs25487 single-nucleotide polymorphism (SNP), which encodes an arginine to glutamine substitution at position 399 (R399Q) was found to be associated with high titer of anti-dsDNA antibodies in Brazilian SLE patients, whereas the presence of two common SNPs was associated with neuropsychiatric manifestations and antiphospholipid syndrome [110]. At the same time, a Chinese Han population cohort study revealed that individuals with the aforementioned SNP are nearly two times more prone to develop SLE compared with healthy controls [111]. Polymorphisms in another key enzyme of BER, Polβ, have also been associated with SLE in two large, independent cohort studies of a Chinese Han population [112,113]. Poly (Adenosine diphosphate-ribose) polymerase 1 (PARP1), a core protein of BER and DSB repair mechanisms, is also implicated in the susceptibility for SLE development. Indeed, a genetic analysis of chromosome 1q41-q42 revealed that a specific allele of PARP1, with a length of approximately 85bp, confers defective DNA repair and abnormal apoptosis, thus predisposing to SLE [114]. The contribution of polymorphisms in DDR/R components to the development and progression of SLE has been recently reviewed here [115].

#### 4.1.2. Increased Endogenous DNA Damage in SLE: Defective Repair or Increased Formation?

We have previously shown that peripheral blood mononuclear cells (PBMCs) from SLE patients display defects in two main DNA repair pathways, namely, NER and DSB repair [3,4]. Specifically, study of the formation of *N*-alkylpurine-monoadducts (almost exclusively repaired by NER) at the N-ras (neuroblastoma RAS) locus, the repair rate of which is representative of the total cellular NER capacity, and phosphorylated H2AX (γ-H2AX), a sensitive marker for DNA DSBs that was measured at the level of the whole cell, revealed that SLE patients with nephritis have approximately 3–5 times higher intrinsic DNA damage compared with healthy controls. Of interest, patients with quiescent disease also exhibited increased levels of DNA damage, although lower than patients with nephritis, suggesting that DNA damage levels may also be associated with disease activity. Following ex vivo treatment of PBMCs with genotoxic drugs such as melphalan or cisplatin, we also observed that SLE patients were defective in NER and DSB repair mechanisms [3,4]. Accordingly, genes involved in NER [DNA damage-binding protein 1 (DDB1), excision repair cross-complementation group 2 (ERCC2), Xeroderma pigmentosum complementation group A (XPA), Xeroderma pigmentosum complementation group C (XPC)] and DSBs repair [Bloom syndrome RecQ like helicase (BLM), checkpoint kinase 1 (CHEK1), HUS1 checkpoint clamp component (HUS1), Meiotic Recombination 11 Homolog A (MRE11A), Nijmegen Breakage Syndrome 1 (Nibrin; NBN), RAD50, RAD51, Replication Protein A1 (RPA1), tumor protein p53 binding protein 1 (TP53BP1), X-ray repair cross complementing 2 (XRCC2), X-ray repair cross complementing 6 (XRCC6)] were significantly downregulated in SLE patients compared with healthy controls [4]. In line with previous data showing that epigenetic dysregulation (particularly global hypomethylation in T cells) is well documented in SLE [116], we also found that SLE patients are characterized by more condensed chromatin structure at the N-ras locus than their matched controls [4]. Moreover, in accordance with previous data showing that the histone deacetylase inhibitor (HDACi) vorinostat reverses the abnormal chromatin compaction that impedes the access of DNA repair proteins to sites of DNA damage, we found that treatment of PBMCs from quiescent SLE patients with this drug resulted in increased efficiency of the DNA repair machinery and decreased DNA damage burden of these cells [4]. Also, B lymphoblastoid cell lines isolated from children with lupus provided adequate information of defects in the repair of DNA DSBs. Indeed, results from neutral comet assay and colony survival assay showed delayed DSBs repair that might contribute further to the progression of autoimmunity, according to the writers [117]. In addition, previous studies have reported that neutrophils from SLE patients are characterized by increased DNA damage, defective repair of oxidative DNA damage, and augmented apoptosis rates [118,119]. Of note, recent data suggest that the enhanced generation of neutrophil extracellular traps (NETosis) driven by mitochondrial ROS promotes externalization of pro-inflammatory oxidized mtDNA and subsequent activation of STING-dependent type I IFN signaling pathway in SLE [120].

In line with our results [4], another research group, which obtained gene expression profiles from SLE patients and healthy individuals, reported downregulation of genes classified in cell cycle sensors (ATPase/ATPase domain-containing genes) and in NER pathway (ERCC2/XPD and ERCC5/XPG) [121]. Indeed, the team concluded that ATP depletion in combination with downregulation of ATP-dependent genes ERCC2 and ERCC5 suggest insufficient DNA repair in SLE patients, resulting in increased apoptosis and perpetuation of autoimmunity. Moreover, in order to identify rare alleles associated with SLE, Delgado-Vega and colleagues performed whole exome sequencing in SLE patients from well-studied Icelandic SLE multi-case families [122]. They found rare, possibly pathogenic variants in 19 genes, including the X-ray repair cross-complementation group 6 binding protein 1 (XRCC6BP1), also termed Ku70-binding protein 3 (KUB3). Of note, the XRCC6 protein, which is involved in NHEJ required for DSB repair pathway and V(D)J recombination, is a well-established lupus autoantigen. Defective PARP1 activity has also been found in PBMCs from SLE patients [123]. In that particular study, Cerboni and colleagues showed that the activity of PARP1 after UV radiation was significantly lower in SLE patients than in healthy controls, suggesting that PARP1 is implicated in the susceptibility for SLE development.

#### 4.1.3. Autoantibodies against DNA Repair Enzymes

The two subunits of Ku protein (Ku70 and Ku80) involved in NHEJ and the DNA-dependent protein kinase (DNA-PK), a pivotal component of the DNA repair machinery that governs the response to DNA damage and is also involved in V(D)J recombination, are known targets of autoantibodies in SLE. Moreover, ELISA and immunoblotting assays in sera from a total of 155 patients with systemic autoimmune diseases identified two more proteins of NHEJ pathway, namely, DNA ligase IV and XRCC4, as autoantibody targets in approximately 20% of SLE patients [124]. Another research group studied by immunoprecipitation the correlation between anti-Ku antibodies in SLE sera and antibodies against four different DNA repair proteins (DNA-PK, PARP, Mre11, and Werner protein) and found that more than 50% of anti-Ku positive sera contained at least one out of four autoantibodies, providing further evidence that abnormal DSB repair influences the development of certain autoimmune diseases [125]. Recently, Luo and colleagues, using a commercial human protein microarray platform bearing over 9400 antigens, compared the autoantibody profile of SLE patients with those of healthy controls and found novel autoantibodies that were related to DNA repair pathways and apoptosis [126]. Interestingly, they observed that the levels of autoantibodies against Apurinic/Apyrimidinic Endodeoxyribonuclease 1 (APEX1), High mobility group box 1 (HMGB1), vaccinia-related kinase 1 (VRK1), Aurora-A kinase (AURKA), peptidyl arginine deiminase 4 (PADI4), and signal recognition particle 19 (SRP19) (all involved in the DDR/R pathways) were positively correlated with the level of anti-dsDNA in SLE patients, suggesting that these autoantibodies may play a critical role in the pathogenesis of SLE.

#### 4.1.4. Defects in Apoptosis and SLE Pathogenesis

Recent studies suggest that either dysregulated apoptosis or defects in dead cell clearance contribute to the perpetuation of autoimmunity and SLE pathogenesis [127]. GWAS studies in the previous years have identified at least eight different genes that function in the clearance of apoptotic cells, finding that their underexpression is related with the development of SLE or a similar phenotype of autoimmunity [128]. Analysis of bone marrow immune cells by immunochemistry from 14 SLE patients (5 of them presented active lupus nephritis at the moment of the bone marrow biopsy) revealed a significantly higher percentage of apoptotic cells than in controls, which was also positively correlated with the number of plasmacytoid dendritic cells, the major type I IFN-a producer [129]. Interestingly, our previous studies have shown that genotoxic drug-induced apoptosis rates were higher in PBMCs from quiescent SLE patients than healthy controls and correlated inversely with DNA repair efficiency, supporting the hypothesis that accumulation of DNA damage contributes to increased apoptosis [3,4]. Also, the same cells after vorinostat treatment showed a suppressed apoptotic rate through modifications in the degree of the chromatin condensation. Accordingly, several apoptosis-associated genes [Protein phosphatase 1 regulatory subunit 15A (PPP1R15A), cyclin-dependent kinase inhibitor 1A (CDKN1A), BRCA1-associated RING domain protein 1 (BARD1), RAD21, RAD9 checkpoint clamp component A (RAD9A), Protein Kinase, DNA-Activated, Catalytic Subunit (PRKDC), Calcium and integrin-binding protein 1 (CIB1), BRCA1, Abelson murine leukemia viral oncogene homolog 1 (ABL1), checkpoint kinase 2 (CHEK2), and Bcl-2-binding component 3 (BBC3)] were found to be significantly overexpressed in SLE compared with healthy controls [4]. Taken together, in these studies we proposed that SLE patients are characterized by lower DNA repair capacity, resulting in the accumulation of DNA damage and the induction of the apoptotic pathway.

Of note, studies in lupus animal models have proven an association between defective apoptosis and SLE development. Indeed, macrophages from mice lacking the intracellular receptor of the membrane tyrosine kinase c-mer (Merkd mice) present in vivo impaired clearance of apoptotic bodies [130]. Moreover, these mice develop a lupus-like autoimmunity phenotype with autoantibodies to ssDNA and dsDNA and renal pathology. In addition, Shao and Cohen reported that mice lacking the T cell immunoglobulin mucin 4 (TIM-4), a phosphatidylserine receptor that assists phagocytosis of apoptotic debris by macrophages, develop autoantibodies to dsDNA (hallmark of SLE), suggesting that this protein is an important component for dead cell clearance [25].

### 4.2. Systemic Sclerosis

Systemic sclerosis is a connective tissue disorder characterized by vascular alterations, autoantibody production, and fibrosis of skin and internal organs [131]. Although the pathophysiology of SSc remains largely unknown, oxidative stress has been implicated in the development and perpetuation of SSc [132]. In fibroblasts isolated from the skin of patients with diffuse SSc, levels of ROS and type I collagen are significantly higher and the amounts of free thiol are significantly lower when compared to normal fibroblasts [133]. Moreover, sera from patients with diffuse SSc and lung fibrosis contain elevated levels of advanced oxidation protein products (AOPPs) compared to sera from healthy individuals or from patients with limited SSc and no lung fibrosis [134]. AOPPS are able to induce hydrogen peroxide production by endothelial cells. Similarly, in vitro treatment of endothelial cells with sera from patients with either limited or diffuse SSc induced higher hydrogen peroxide production compared to sera from healthy individuals [134]. AOPPs are also able to induce proliferation of fibroblasts. Of interest, in vitro synthesized AOPPs, from DNA topoisomerase 1 oxidized by hypochlorous acid or hydroxyl radicals, increased the proliferation of fibroblasts and the production of hydrogen peroxide by endothelial cells compared to AOPP generated from other proteins [134]. In line with these results, oxidative stress induced by either immunoglobulins isolated from SSc patients or by oxidative DNA-damaging agents led to decreased Wingless inhibitory factor 1 (WIF-1) expression in SSc fibroblasts and was associated with higher collagen production. This effect, mediated by the central DDR kinase ATM, linked oxidative stress and DNA damage with fibrosis, suggesting an important role of the DDR/R pathway in pathogenesis of fibrosing conditions such as SSc [135]. On the other hand, inhibition of ATM in SSc fibroblasts with the competitive inhibitor KU55933 (KuDOS 55933) significantly increased the expression of the WIF-1 gene, suggesting a therapeutic benefit from targeting components of the DDR/R [135].

Moreover, increased DNA damage levels have also been detected in the peripheral blood of patients with SSc, regardless of disease subtype (diffuse or limited SSc) or treatment [23]. To examine whether DNA damage is a result of dysfunction of DNA repair enzymes, DNA damage and polymorphic sites in two genes encoding DNA repair enzymes XRCC1 [arginine to glutamine polymorphism at position 399 (Arg399Gln)] and XRCC4 [Isoleucine to threonine polymorphism at position 401 (Ile401Thr)] were evaluated. Regarding the XRCC1 gene, healthy individuals with the Arg399Gln allele presented higher levels of DNA damage compared with healthy individuals with the XRCC1 wild type, something that was not observed in SSc patients. However, SSc patients with either XRCC1 allele presented increased DNA damage compared to healthy individuals. Regarding the XRCC4 gene, both healthy individuals and SSc patients with the Ile401Thr allele presented higher levels of DNA damage compared to healthy individuals or SSc patients with the XRCC4 wild type allele [23]. Together, these results indicate that SSc patients with polymorphisms at genes of DNA repair enzymes are characterized by increased DNA damage. Of interest, XRCC4 was also found to be enriched in patients with diffuse SSc in a study using whole-exome sequencing (WES) in 32 diffuse cutaneous systemic sclerosis (dcSSc) patients and 17 healthy controls [136].

### 4.3. Rheumatoid Arthritis

The role of oxidative DNA damage and aberrations of the DDR/R network have been long studied in RA [137]. Initial studies reported overexpression and tissue-specific mutations of p53, a central molecule in DNA repair and regulator of apoptosis, in the synovium of patients with RA [138,139]. P53 mutations were characteristically detected at the lining region of the synovium [140], which mainly consists of fibroblast-like synoviocytes (FLS), the “maestro” of synovial inflammatory milieu [141], and macrophage-like synoviocytes. Immunohistochemical analysis of RA synovial tissues revealed compensatory up-regulation of MMR enzymes, especially in the synovial lining, which, however, did not completely invert the observed oxidative damage [142]. Of interest, neutrophils isolated from synovial fluid of RA patients also displayed increased DNA damage levels when compared to osteoarthritic controls [143]. Extracellular mitochondrial DNA and 8-oxo-2′-deoxyguanosine (8-oxodG) DNA was also detected in synovial fluid from RA patients but not in controls [144].

Further, we and others have detected increased endogenous DNA damage levels in peripheral blood (PBMCs or granulocytes) of patients with RA compared with healthy controls [24,89,143]. Of interest, a positive correlation of endogenous DNA damage levels in PBMCs/peripheral blood neutrophils with the disease activity index DAS-28 has also been observed [89,143]. Previous studies have reported lower levels of DNA damage in neutrophils and T cells of patients under treatment compared with treatment-naïve patients. [143,145]. In line with these results, in our recent study we examined paired samples from patients before and after 12 week antirheumatic treatment and observed a significant decrease in the endogenous DNA damage levels [24].

Accumulation of the endogenous DNA damage in cells can be mediated either by augmented endogenous DNA damage formation and/or delayed/decreased efficiency of the DNA repair mechanisms, two possibilities that are not mutually exclusive. Numerous studies have shown increased levels of oxidative stress in RA PBMCs/neutrophils in correlation with endogenous DNA damage levels [24,89,143]. Increased levels of 8-oxodG have also been found in DNA of peripheral blood lymphocytes, CD4+ T cells, and granulocytes of RA patients [145,146]. Further, we have recently shown that abasic site formation, the most common spontaneously occurring DNA lesion, is also increased in patients with RA [24].

On the other hand, previous studies have shown significant defects in the DNA repair capacity of RA patients in association with increased senescence and apoptosis [145,146]. When cultured, RA lymphocytes showed increased apoptotic rates in association with spontaneous accumulation of DNA damage [145], whereas they were also more sensitive to hydrogen peroxide-induced DNA damage and growth-arrest [146]. Further, RA T cells were more sensitive to ionizing radiation and showed delayed repair of DNA damage. Several sensors of DSBs were down-regulated in RA T cells (ATM, Rad50, MRE11, and NBS1) in the basal state and also failed to increase in response to radiation-induced DNA damage [145]. Of note, recent studies have shown that MRE11A, in addition to its DNA repair activity, plays a critical role in mitochondria protection, as the deficiency of MRE11A in RA T cells disrupted mitochondrial oxygen consumption; suppressed ATP generation; caused leakage of mitochondrial DNA into the cytosol; and induced inflammasome assembly, caspase-1 activation, and pyroptotic cell death [147]. In line with these results, we have recently shown that RA PBMCs are characterized by decreased repairing capacity, mainly due to defects in the global genome repair (GGR) pathway of NER, directly controlled by the degree of chromatin condensation, and we also showed that 3 month treatment with antirheumatic drugs reversed the observed phenotype [24].

Taken together, accumulation of endogenous DNA damage, derived from augmented formation of DNA damage and deregulated DDR/R signals, which are both reversible after therapy, is implicated in the pathogenesis of RA. Last but not least, although Shao and colleagues [145] failed to spot similar deficiencies in components of the DNA repair machinery in SLE-derived CD4+ T cells, we have previously shown strong down-regulation of both ATM and MRE11 complex in PBMCs of patients with quiescent SLE [4]. The possibility of a shared defective mechanism among autoimmune diseases is rather tempting, taking into consideration the central role of nucleic acid metabolism and recognition in initialization and perpetuation of autoimmunity (Figure 1) [22].

## 5. Conclusion and Future Directions

**Figure 2:**
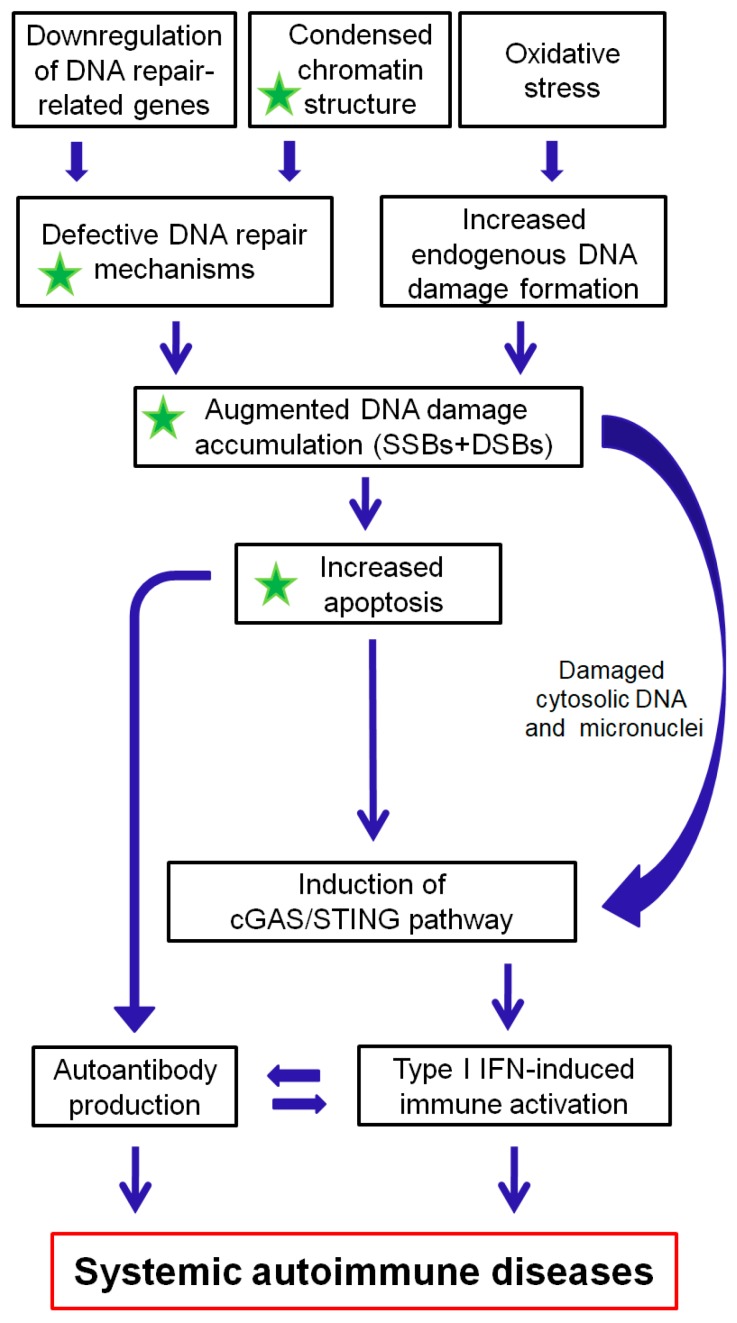
A proposed model of systemic autoimmune disease promotion by epigenetically regulated functional abnormalities of the DNA damage response and repair (DDR/R) network and oxidative stress. The green asterisk denotes partial reversibility following histone hyperacetylation. SSBs: single-strand breaks, DSBs: double-strand breaks.

The DDR/R network and the ImmR act synergistically for the survival of all living organisms. Aberrant activation of each one of these systems often leads to chronic and potentially fatal systemic autoimmune diseases. As reviewed herein, a balance shift in DDR/R may negatively affect ImmR; evidently, the opposite also may occur. As depicted in Figure 2, we propose that epigenetically regulated functional abnormalities of DNA repair mechanisms (i.e., downregulation of DDR/R-related genes and condensed chromatin structure that result in defective repair) and increased endogenous DNA damage formation, partly due to the induction of oxidative stress, may result in the augmented accumulation of DNA damage (both SSBs and DSBs). This accumulation may trigger the induction of apoptosis, which facilitates autoantibody production, as well as the generation of damaged cytosolic DNA and micronuclei that both can act as potent immunostimulators through the induction of the cGAS-STING-IRF3 pathway and the production of type I IFN, leading to systemic autoimmune disease expression. Notably, some of the components are partially reversible following histone hyperacetylation.

Because targeting the DDR/R network can have an impact not only on cancer cells but also on host immunity, manipulation of molecular components of this network, alone or in combination with immune checkpoint inhibitors, has gained significant attention in cancer immunotherapy [148,149]. Although these approaches have been extensively studied in cancer, promising results have also been revealed in preclinical mouse models of autoimmunity. Therapeutic targeting of T lymphocytes from autoimmune disease patients with DDR/R inhibitors is based on their high proliferation rate and accumulation of DNA damage [150]. Indeed, in mice with experimental autoimmune encephalitis, the combination of p53 activators and CHK1/2 inhibitors led to the elimination of pathogenic, activated T lymphocytes with no side-toxicity of normal T cells. In addition, our recent data have shown that treatment of human SLE-derived PBMCs with the HDACi vorinostat results in hyperacetylation of histone H4, chromatin decondensation, restoration of the DNA repair capacity, and decreased apoptosis rates [4]. These results are in line with previous data, showing that HDACi ameliorate disease in lupus mouse models [151,152,153]. Also, treatment of lupus-prone Mrl/lpr mice with the HDACi panobinostat significantly reduced circulating naïve B and plasma cell numbers and the levels of autoantibodies [154]. More importantly, in children with systemic-onset juvenile idiopathic arthritis, the HDACi givinostat was found to be safe and beneficial, particularly in reducing the arthritic features, suggesting that HDACi may have important clinical applications in the treatment of systemic autoimmunity [155]. On the other hand, restoration of defective DNA repair factors, such as MRE11A, has also shown promising results in reducing the pro-inflammatory, pro-arthritogenic capacity of RA T-cells in vivo [156], whereas ATM overexpression in RA T cells was able to invert the observed apoptotic phenotype [145].

Taken together, the results reviewed herein suggest that the deregulated interplay between DDR/R and ImmR plays a crucial role in the pathogenesis and progression of systemic autoimmune diseases. Thus, unraveling the molecular mechanisms of this interplay can be exploited for understanding pathogenesis and progression of these diseases, as well as to discover new treatment opportunities in the field.
